# Sodium-Dependent Neutral Amino Acid Transporter 2 Can Serve as a Tertiary Carrier for l-Type Amino Acid Transporter 1-Utilizing Prodrugs

**DOI:** 10.1021/acs.molpharmaceut.2c00948

**Published:** 2023-01-23

**Authors:** Johanna Huttunen, Thales Kronenberger, Ahmed B. Montaser, Adéla Králová, Tetsuya Terasaki, Antti Poso, Kristiina M. Huttunen

**Affiliations:** † School of Pharmacy, Faculty of Health Sciences, University of Eastern Finland, P.O. Box 1627, FI-70211 Kuopio, Finland; ‡ Department of Internal Medicine VIII, University Hospital Tübingen, Otfried-Müller-Strasse 14, DE 72076 Tübingen, Germany; § Department of Pharmaceutical and Medicinal Chemistry, Institute of Pharmaceutical Sciences, Eberhard-Karls-Universität, Tübingen, Auf der Morgenstelle 8, 72076 Tübingen, Germany; ∥ Cluster of Excellence iFIT (EXC 2180) “Image-Guided and Functionally Instructed Tumor Therapies”, University of Tübingen, 72076 Tübingen, Germany; ⊥ Tübingen Center for Academic Drug Discovery & Development (TüCAD2), 72076 Tübingen, Germany

**Keywords:** l-type amino acid transporter 1 (LAT1), membrane transporters, molecular dynamics (MD) simulation, prodrugs, sodium-coupled neutral amino acid transporter 2 (SNAT2)

## Abstract

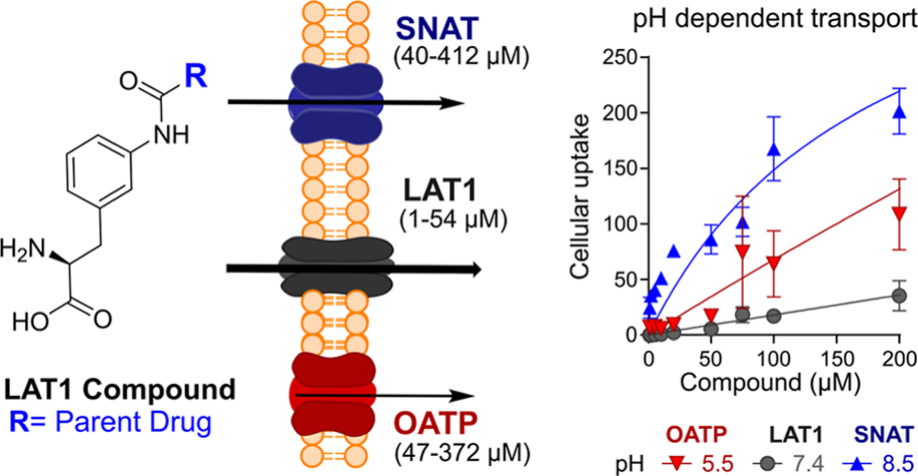

Membrane transporters are the key determinants of the homeostasis of endogenous compounds in the cells and their exposure to drugs. However, the substrate specificities of distinct transporters can overlap. In the present study, the interactions of l-type amino acid transporter 1 (LAT1)-utilizing prodrugs with sodium-coupled neutral amino acid transporter 2 (SNAT2) were explored. The results showed that the cellular uptake of LAT1-utilizing prodrugs into a human breast cancer cell line, MCF-7 cells, was mediated via SNATs as the uptake was increased at higher pH (8.5), decreased in the absence of sodium, and inhibited in the presence of unselective SNAT-inhibitor, (α-(methylamino)isobutyric acid, MeAIB). Moreover, docking the compounds to a SNAT2 homology model (inward-open conformation) and further molecular dynamics simulations and the subsequent trajectory and principal component analyses confirmed the chemical features supporting the interactions of the studied compounds with SNAT2, which was found to be the main SNAT expressed in MCF-7 cells.

## Introduction

Biological membranes or biomembranes consist of, in addition to phospholipid bilayer, a wide variety of membrane receptors and proteins as well as glycoproteins, glycolipids, and cholesterol.^1^ Many transmembrane proteins, such as ion channels, ATPases, solute carriers (SLCs), and ATP-binding cassettes (ABCs), function as gateways for endogenous and exogenous compounds, permitting their cargo either into the cells (import or influx) or out of the cells (export or efflux).^2^ SLC, with nearly 500 protein members, is the largest family of transporters encoded by the human genome, but at the same time, they are also the less studied and exploited group of proteins in drug discovery and development.^3,4^ Although some of the members of SLC are intensively studied and successfully utilized as drug targets, many of SLC members are still orphans; their biological function and substrate specificities are not well known.

Transporters are known to greatly affect not only the endogenous homeostasis of the cells but also the pharmacokinetic/pharmacodynamic profiles of many drugs as well as drug–drug interactions. Therefore, mutations, polymorphism, and up- or down-regulation of these proteins should be characterized more thoroughly since SLCs may be the key players in drug resistance or treatment failures.^2,5^ More importantly, it has been acknowledged that at least half of the SLCs are linked to human diseases, including cancer, inflammatory diseases, diabetes, and mental disorders, to name a few.^6^ Currently (in 2022), U.S. Food and Drug Administration (FDA) and European Medical Agency (EMA) define only 10 transporters (2 ABCs and 8 SLCs) and thus less than 2% of them all, whose interactions should be evaluated during the drug discovery and development. Since it is known that the tissue expression and function of SLCs can be regulated simply by the physiological state of the cells,^7^ more efforts should be paid to unreveal the medical relevance and therapeutic significance of SLCs.

Although it has been recognized that a single molecule can be a substrate or inhibitor for several transporters, there has been surprisingly little focus on how it affects the pharmacokinetics or pharmacodynamics of drugs and subsequently the toxicity or drug–drug interactions. This is mainly because we lack proper methods to study transporters,^8,9^ and we usually are interested only in one target transporter. This may lead to misinterpretations and a lack of clinical success. Our research group has developed l-type amino acid transporter 1 (LAT1)-utilizing prodrugs for brain-targeting purposes for over a decade^10−19^ since LAT1 is highly expressed particularly at the blood–brain barrier and brain parenchymal cells.^20^ We have also reported that most of the LAT1-utilizing prodrugs bear a low affinity for organic anion-transporting polypeptides (OATPs), which may have crucial effects if LAT1 is saturated or becomes dysfunctional on the plasma membrane due to the disease, diet, or environmental factors.^11,21^

The involvement of OATPs in the total uptake of LAT1-utilizing prodrugs was initially discovered in a slightly acidic environment (pH 5; one point concentration), in which OATP-mediated transport of substrates is particularly high.^21^ In the present study, we were able to discover another, tertiary transport mechanism that was increased in mildly alkaline conditions (pH 8.5). We hypothesize that the transporter(s) that can carry LAT1-utilizing prodrugs belong to the sodium-coupled neutral amino acid transporter (SNAT) family, which presents one of the few transporters that are activated in elevated and slightly alkaline pH.^8^

SNATs 1–11 (*SLC38A1–11*) are transmembrane proteins that mediate the cellular uptake of neutral amino acids in sodium- and pH-dependent manners.^22−24^ SNAT1–5 and 7 are relatively well characterized, while the rest of the family is still in their early evaluation. Moreover, the old classification of system A (SNAT1, 2, and 4; Na^+^-dependent electrogenic transport with 1:1 stoichiometry), also known as SATs, and system N (SNAT3, 5, and 7; electroneutral Na^+^/H^+^ antiporters), also recognized as SNs, confuses the prevailing terminology, particularly with the less studied SNAT members. However, these two classes transport different amino acids; for example, system A members with a broader substrate specificity carry substrates, such as l-forms of methionine, proline, serine, glutamine, asparagine, histidine, and arginine, while system N members with more narrow transport profiles favor l-forms of histidine, asparagine, and glutamine; some of them (SNAT7) also favor alanine and serine.^25,26^ SNATs are relatively widely distributed throughout the body; however, SNAT1–7 have also been found in the brain and more specifically in the neurons (SNAT1, 2) as well as in astrocytes (SNAT3 and 5).^24,27−30^ This means that if they can interact with LAT1-utilizing compounds, they may increase the total brain uptake and accumulation of these compounds in the parenchymal cells.

Therefore, we evaluated herein the cellular uptake of eight LAT1-utilizing prodrugs (**1–8**; Table 1) in different conditions in a human breast cancer cell, MCF-7, that is known to express SNAT1–2, 6–7, 9–10 according to the human protein atlas (www.proteinatlas.org, accessed 9.5.2022). In addition, the cellular uptake of two other known LAT1 substrates, thyroxin (T_4_) and l-Trp, OATP-substrate probenecid (PRB; negative control), and an unselective system A (SNAT1–2, 4) ligand or so-called inhibitor, MeAIB [α-(methylamino)isobutyric acid, positive control],^31−33^ was carried out and compared. Thus, this study will give insights into possible tertiary interactions of LAT1-utilizing transporters not only with OATPs but also with SNATs, which may affect their system pharmacokinetics as well as targeting purposes, for example, into the brain.

**Table 1:**
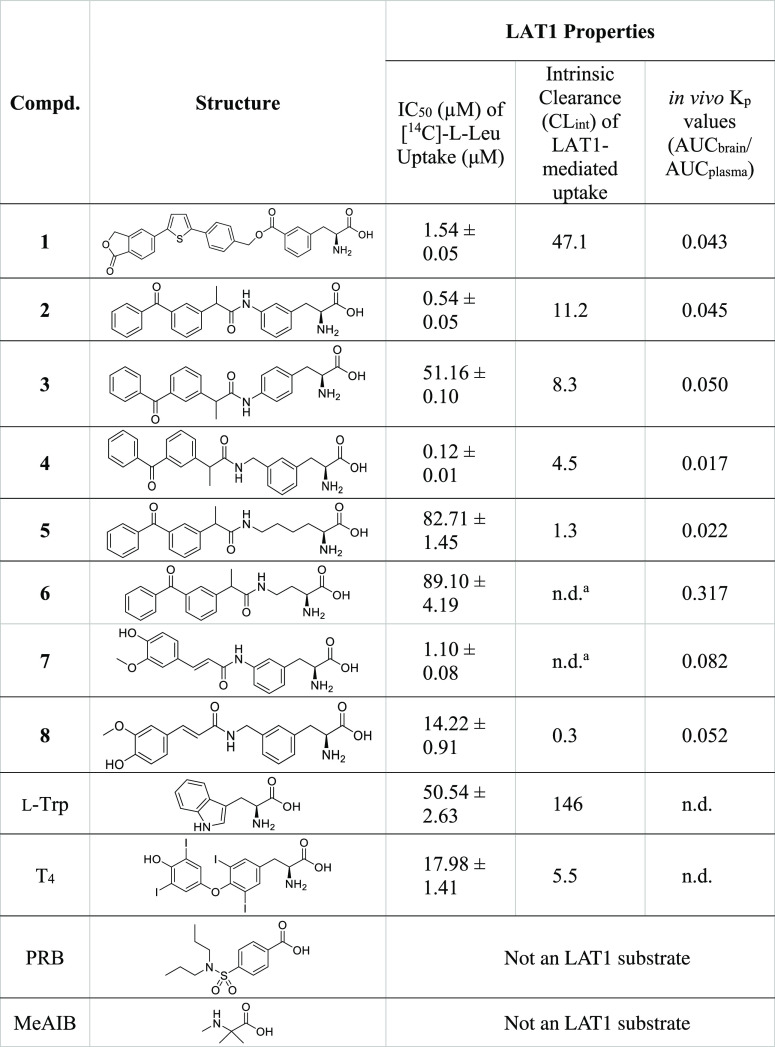
Structures of Studied Compounds; LAT1-Utilizing Prodrugs **1–8**, LAT1 Substrates, Thyroxin (T_4_) and l-Trp, OATP-Substrate Probenecid (PRB, Negative Control), and an Unselective System A (SNAT1–2,4) Ligand MeAIB [α-(Methylamino)-isobutyric Acid, Positive Control] and Their LAT1-Substrate Properties [Ability to Compete with Natural LAT1-Substrate l-Leu Presented as Half Maximal Inhibitory Concentration IC_50_ Values and Transport Efficiency via LAT1 Presented as Intrinsic Clearance (CL_int_ = *V*_max_/*K*_m_)] and Brain Accumulation (Presented as *K*_p_ Values = AUC_brain_/AUC_plasma_ Ratio) as Reported Previously^15,18,19,21^

a n.d. = not determined, due to the insufficient data points.

## Materials and Methods

### Chemicals

All reagents and solvents used in analytical studies were commercial and with high purity and of analytical grade or ultra-gradient HPLC grade purchased from MilliporeSigma (St. Louis, MO, USA), Thermo Fisher Scientific (Waltham, MA, USA), J.T. Baker (Deventer, The Netherlands), Riedel-de Haën (Seelze, Germany), EuroClone S.p.A. (Pero, Italy), or Promega Biotech AB (Nacka, Sweden). Unlabeled and stable-isotope-labeled peptides used to quantify target proteins were kindly provided from Tohoku University with a Material Transfer Agreement between Tohoku University (Japan) and University of Eastern Finland. Water was purified using a Milli-Q Gradient system (Millipore, Milford, MA, USA). Synthesis, structural characterization [^1^H NMR, ^13^C NMR, liquid chromatography–mass spectrometry (LC–MS)], over 95% purity (elemental analysis), and the LAT1-mediated transport of the studied prodrugs (Table 1) have been reported earlier for compound **1**,^11^ compounds **2–6**,^18^ and compounds **7–8**.^19^

### Biological Material

MCF-7 human breast adenocarcinoma cells (HTB-22) were purchased from the American Type Culture Collection (ATCC, Manassas, VA, USA). MCF-7 cells were cultured in Dulbecco’s modified Eagle’s medium supplemented with l-glutamine (2.0 mM), heat-inactivated fetal bovine serum (10%), and penicillin (50 U/mL)–streptomycin (50 μg/mL) solution. MCF-7 cells (passages 8–25) were seeded at the density of 1 × 10^5^ cells/well onto 24-well plates. The cells were used for the uptake experiments 1 day after seeding. All the studies were carried out as three biological replicates from the same cell passage. The function of SNATs was followed between the used cell passages with a SNAT probe substrate, [^3^H]-l-proline, and noticed to be unaltered.

### Expression of SNATs in MCF-7 Cells

The absolute expressions of SNATs were quantified from the plasma membrane fractions of MCF-7 cells by LC–MS/MS method following a multiplexed multiple reaction monitoring (MRM) analysis mode according to the protocol described earlier^34^ with minor modifications.^35−37^ The plasma membrane fractions were isolated from three distinct sets of cell culture plates (biological replicates) by using a Membrane Protein Extraction Kit (BioVision Incorporated, Milpitas, CA, USA) according to the manufacturer’s instructions. The protein content for each fraction was measured using a Pierce BCA Protein Assay Kit (Thermo Fisher Scientific, Inc., Waltham, MA, USA), and a total amount of 50 μg proteins from each fraction was denatured, reduced, and alkylated. Finally, the peptides in the precipitated protein pellet were digested with LysC (1/100, w/w) and 0.05% ProteaseMax for 3 h at room temperature. The samples were spiked with 10 μL (30 fmol) of the labeled peptides for absolute quantification (Table S1) and further digested with TPCK-Trypsin (1/100, w/w; Promega Biotech AB, Nacka, Sweden) for 18 h at 37 °C, followed by acidification. The digested peptides in each sample were analyzed by using an ultra-performance liquid chromatography system coupled with a triple-quadrupole mass spectrometer with a heated electrospray ionization source in the positive mode (UPLC 1290 and MSD 6495, Agilent Technologies, Santa Clara, CA, USA). A total amount of 20 μL of the digested peptides (10 μg) was separated using an AdvanceBio Peptide Map 2.1 × 250 mm, 2.7 μm column (Agilent Technologies, Santa Clara, CA, USA), eluting with of 0.1% formic acid in water (A) and acetonitrile (B) with a constant flow rate of 0.3 mL/min and a gradient of 2–7% B for 2 min, followed by 7–30% B for 48 min, 30–45% B for 3 min, and 45–80% B for 2.5 min before re-equilibrating the column again for 4.5 min. The proteins were quantified based on the ratio between the light and heavy standard peptides, as described previously (Table S1).^36^ Data were acquired using Agilent MassHunter Workstation Acquisition (Agilent Technologies, Data Acquisition for Triple Quadrupole, version B.03.01) and processed by using Skyline software (version 20.1). The results were compared to a housekeeping protein Na^+^/K^+^ATPase and expressed as fmol/μg of the total amount of protein in the samples.

### Functionality of SNATs in MCF-7 Cells

After removal of the culture medium, MCF-7 cells were carefully washed with prewarmed HBSS (Hank’s balanced salt solution) containing 125.0 mM NaCl (or choline chloride in Na^+^-free conditions), 4.8 mM KCl, 1.2 mM MgSO_4_, 1.2 mM KH_2_PO_4_, 1.3 mM CaCl_2_, 5.6 mM glucose, and 25.0 mM 4-(2-hydroxyethyl)piperazine-1-ethanesulfonic acid (HEPES) with pH adjusted to either 7.4 or 8.5 with 1 M NaOH (or KOH in sodium-free conditions). In the experiments at lower pH (4.5–6.5), 25.0 mM HEPES was replaced by 2-(*N*-morpholino)ethanesulfonic acid (MES), and pH was adjusted to 4.5, 5.5, and 6.5 by 1.0 M NaOH. Preincubation was done with 500 μL of prewarmed HBSS at 37 °C for 10 min before adding substrates (250 μL in HBSS) for the uptake experiments. The functionality of SNATs in MCF-7 cells was studied with a known SNAT substrate; the cells were incubated either with [^3^H]-l-proline [1 μM (PerkinElmer, Waltham, MA, USA) in uptake buffer HBSS, 250 μL] at 37 °C for 1–60 min or with the uptake buffer (250 μL) consisting of 1–600 μM l-proline (containing 2 μCi [^3^H]-l-proline) at 37 °C for 60 min.^38^ After incubation, the uptake was stopped by adding 500 μL of ice-cold HBSS, and the cells were washed two times with ice-cold HBSS (2 × 500 μL). The cells were then lysed with 500 μL of 0.1 M NaOH (60 min), and the lysate was mixed with 3.5 mL of Emulsifier safe cocktail (Ultima Gold, PerkinElmer, Waltham, MA, USA). The radioactivity in the cells was measured by liquid scintillation counting (MicroBeta^2^ counter, PerkinElmer Waltham, MA, USA). The concentrations of [^3^H]-l-proline were calculated from the spiked standard curve at each pH and normalized with the protein concentrations. The SNAT-mediated uptake was confirmed by changing the pH of the uptake buffer in the absence of sodium ions and in the presence of unselective SNAT-ligand, MeAIB (100 μM).^31,32^

### Transporter-mediated Uptake of Compounds into Cells

For the following experiments, the MCF-7 cells were cultured, seeded, washed with HBSS, and preincubated as described above at the desired conditions (pH 5.5, 7.4, or 8.5, with and without Na^+^). Cellular uptake of prodrugs **1–8**, l-Trp, T_4_, and PRB was then studied by adding compounds at the concentration of 1–200 μM in prewarmed HBSS buffer (250 μL) on the cell layer and incubating at 37 °C for 30 min (uptake was linear with all compounds up to 30 min). Subsequently, the cells were washed three times with ice-cold HBSS and lysed with 500 μL of 0.1 M NaOH (60 min). The supernatants were analyzed by the LC–MS/MS methods described earlier for compound **1** (perforin inhibitor prodrug),^15,19^ compounds **2–6** (ketoprofen prodrugs),^18^ and compounds **7–8** (ferulic acid prodrugs)^19^ with an Agilent 1200 Series Rapid Resolution LC System together with an Agilent 6410 Triple Quadrupole Mass Spectrometer equipped with an electrospray ionization source using a Zorbax XDB-C18 Eclipse Rapid Resolution High Throughput column (50 mm × 4.6 mm, 1.8 μm; Agilent Technologies, Santa Clara, CA) for the liquid chromatographic separation of the analytes. The lower limit of quantification for compound **1** was 0.5 nM, and for compounds **2–8**, l-Trp, T_4_, and PRB, it was 0.05 nM. These LC–MS/MS methods were also selective, accurate (100 ± 10%), and precise (RSD < 10%) over the range of 1.0–100 nM. The cell-associated concentrations of each compound normalized to protein concentration were calculated from the standard curve that was prepared by spiking known concentrations of compounds to ACN including the selected internal standard (diclofenac to all studied compounds). The protein concentrations on each plate were determined as a mean of three samples by Bio-Rad Protein Assay based on the Bradford dye-binding method using BSA as a standard protein and measuring the absorbance (595 nm) by a multiplate reader (EnVision, PerkinElmer, Inc., Waltham, MA, USA).

The competitive uptake in the presence of unselective SNAT ligand, MeAIB (100 μM),^31,32^ was carried out as described above with HBSS buffer at pH 7.4 or pH 8.5 containing 100 μM of the studied compound. The cells were preincubated with the inhibitors for 10 min, and the incubation mixture was removed before adding the studied compound and the inhibitor to the cells. The competitive uptake (30 min) with the inhibitor was then carried out as the normal uptake described above. The concentrations of studied compounds were analyzed by the LC–MS/MS method and calculated from the spiked standard curve and normalized with the protein concentrations.

### Homology Model and Proposed Binding Mode of the Compounds

SNAT2 3D-structure model was derived from the AlphaFold database (SLC38A2 UniProt ID: Q96QD8, AlphaFold identifier: AF-Q96QD8-F1),^39,40^ from which the first 65 amino acids were removed due to the very low-quality prediction. The model was prepared and minimized by adding hydrogens, adjusting protonation states of amino acids, and fixing missing side-chain atoms and protein loops by using Maestro PrepWizard 2021.4.

All ligands for docking were drawn using Maestro (2021.4) and prepared using LigPrep to generate the three-dimensional conformation, adjust the protonation state to physiological pH (7.4), and calculate the partial atomic charges, with the force-field OPLS4. All ligands that could generate racemic mixtures were studied in the (s,s) configuration (the amino acid like) with exception of compound **6**, which was simulated in both (s,s) and (s,r). We employed induced-fit docking to accommodate the unselective SNAT ligand, MeAIB, and used the best scoring coordinate of this compound as a reference to guide the docking of all the other studied compounds **1**, **5**, **6**, **8**, and T_4_ within the SNAT’s ligand-binding pocket using Glide.^41^ Ligands were docked within a grid around 12 Å from the centroid of the predicted binding pocket. The binding site was predicted by superimposing the generated model with the l-arginine-bound *Dr*SLC38A9 structure (PDB ID: 6C08), where the conserved aminoacid portions guided the pocket selection. For the best scoring pose of each mentioned ligand, a system with different pHs (5.5, 7.4, and 8.5) was generated using Epik,^42^ also implemented in PrepWizard 2021.4.

### Molecular Dynamics Simulations and Trajectory Analyses

The minimized structures were submitted to molecular dynamics (MD) simulations for further refinement. Selected docking poses were further validated by MD simulations, where ligand stability within the proposed pocket and its interactions were evaluated. The MD simulations were carried out using the Desmond engine^43^ with the OPLS4 force-field.^44^ The simulated system encompassed the protein–ligand complex, a predefined water model (TIP3P^45^) as a solvent, POPC membranes (automatically positioned according to the α-helices), and counterions (Na^+^ or Cl^–^ adjusted to neutralize the overall system charge). The system was treated in an orthorhombic box with periodic boundary conditions specifying the shape and the size of the box as 10 × 10 × 13 Å distance from the box edges to any atom of the protein. RESPA integrator time steps of 2 fs for bonded and near and 6 fs for far were applied. Short-range Coulombic interactions were performed using a time step of 1 fs and a cutoff value of 9.0 Å, whereas long-range Coulombic interactions were handled using the (smooth particle mesh Ewald method^46^). Standard Desmond relaxation protocol was employed. Simulations were run in the *NPT* ensemble, with a temperature of 310 K (Nosé–Hoover thermostat) and a pressure of 1.01325 bar (Martyna–Tobias–Klein barostat).

The results of simulations, in the form of trajectory and interaction data, are available on the Zenodo repository (codes: 10.5281/zenodo.6538694). MD trajectories were visualized, and figures were produced using PyMOL v.2.5 (Schrödinger LCC, New York, NY, USA). For each ligand, simulations at least three independent 200 ns replicas were carried out, with compound **1** being simulated for 3 × 500 ns, resulting in 15 μs worth of simulations for all the simulated systems.

Protein–ligand interactions were determined using the simulation event analysis pipeline implemented in Maestro (Maestro v2021.4). Distance calculations were performed employing the Maestro event analysis tool (Schrödinger, LLC, New York, NY). Distances between specific secondary structure elements were calculated using their centers of mass, using the script trj_asl_distance.py having as an argument the atom numbers of the residues involved in the interaction.

### Principal Component Analysis

Extreme motions of the protein complexes during the MD simulations were analyzed using principal component analysis (PCA). All the python scripts used in this study were provided by Schrödinger. Analysis was run considering only the variation of the backbone atoms from the entire transporter, which were kept using trj_keep_selection_dl.py script. The entire trajectory was then aligned to frame 0 (initial frame) using trj_align.py script, and trajectories from all the simulations were merged using the python script trj_merge.py. The combined trajectory was used to generate .xtc and .pdb files (required for the Gromacs software) using trj_no_virt.py script, followed by our in-house developed script (fix_pdb.py) used to fix the pdb file generated in the previous step.

The generated files were used for PCA by using GROMACS tools (version 2020). Further, PCA was carried out by using gmx anaeig following the covariance matrix generation using the gmx covar command line, with standard options. Extreme motion figures were generated and visualized using modevectors script from PyMOL.^47^

### Structure and Data Visualization

Structure visualization was conducted with PyMOL v.2.5 (Schrödinger LLC, New York, NY, USA). Data visualization was completed by Python 3.7, seaborn, and matplotlib.^48,49^

### Data Analysis

All data analyses, including Michaelis–Menten and Eadie–Hofstee analyses, were performed using GraphPad Prism v. 5.03 software (GraphPad Software, San Diego, CA, USA). Statistical differences between groups were tested using one-way ANOVA, followed by a two-tailed Tukey’s multiple comparison test, and presented as mean ± SD, with statistically significant differences denoted by *P < 0.05, **P < 0.01, ***P < 0.001, and ****P < 0.0001.

## Results

### Expression and Function of SNATs in MCF-7 Cells

**Figure 1:**
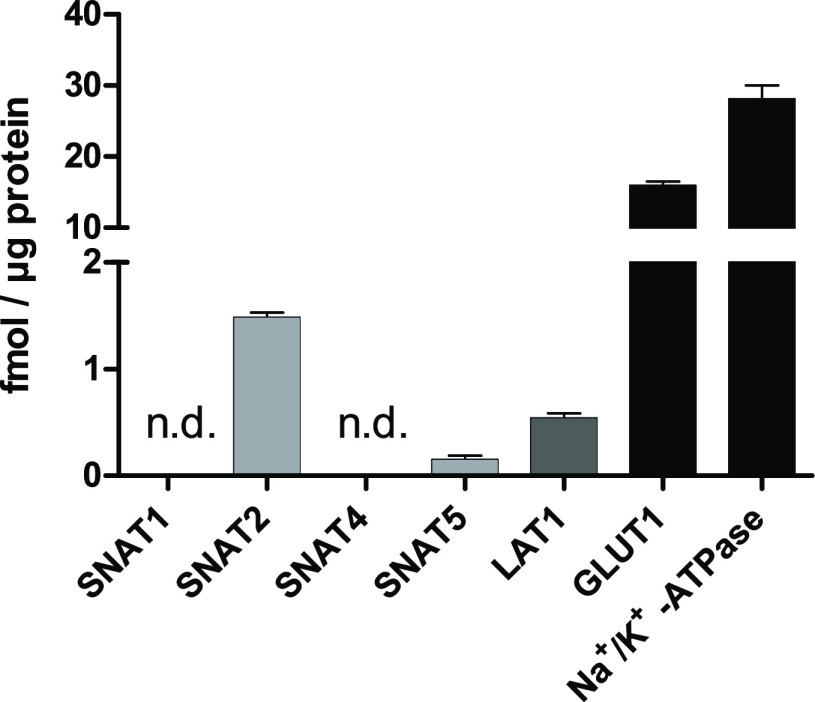
Quantitative protein levels of SNATs 1, 2, 4, and 5 together with LAT1, GLUT1, and sodium-potassium adenosine triphosphatase (Na^+^/K^+^-ATPase) analyzed from the plasma membranes of human breast cancer cells, MCF-7, normalized to the total amount of protein in the plasma membrane. The results are expressed as mean ± SD (*n* = 3; biological replicates), and n.d. denotes not detected.

The human estrogen receptor-positive breast adenocarcinoma cell line, MCF-7 (Michigan Cancer Foundation-7), was used in the present study as we have recently reported the expression and function of LAT1 in this cell line.^21^ Here, we quantified first the protein expression of SNAT1, SNAT2, SNAT4, and SNAT5 from the plasma membrane fractions with LC–MS/MS methods. The protein amounts of SNATs are compared to the expression of LAT1, glucose transporter 1 (GLUT1), which are highly expressed in many cancer cells, and sodium-potassium adenosine triphosphatase (Na^+^/K^+^ATPase), which is regarded as a housekeeping protein expressed highly on the plasma membranes. As seen in Figure 1, the expression level of SNAT2 was the highest (1.48 ± 0.05 fmol/μg protein) of all studied (SNAT1 and SNAT4 were not detected at all, and the expression level of SNAT5 was 0.15 ± 0.04 fmol/μg protein). Curiously, the expression of SNAT2 was almost 3 times greater than that of LAT1 (0.54 ± 0.04 fmol/μg protein) but 10 times smaller than that of GLUT1 (15.9 ± 0.6 fmol/μg protein) and nearly 20 times smaller than that of Na^+^/K^+^ATPase (28.1 ± 2.0 fmol/μg protein).

**Figure 2:**
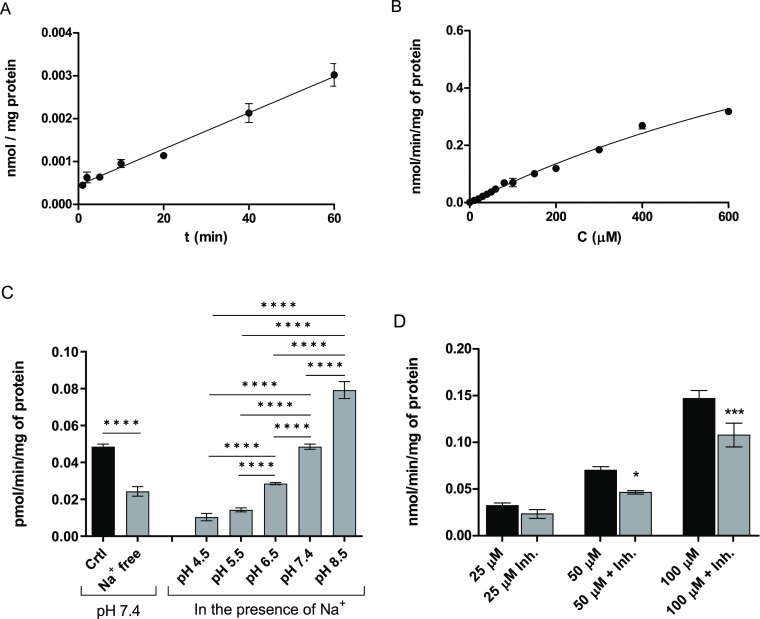
Cellular uptake of [^3^H]-l-proline; (A) time-dependent uptake (1 μM within 1–60 min), (B) concentration-dependent uptake (10–600 μM, during 60 min), (C) sodium-dependent uptake at pH 7.4 (left) and pH (4.5–8.5)-dependent uptake in the presence of Na^+^ (right), and (D) uptake (25, 50, and 100 μM) in the presence of an unselective SNAT ligand, MeAIB (100 μM). Data are presented as mean ± SD, *n* = 3, and an asterisk denotes a statistically significant difference from the respective control uptake (black bars) (*P < 0.05, ***P < 0.001, and ****P < 0.0001, one-way ANOVA, followed by Tukey’s multiple comparison test).

The function of SNATs was demonstrated with a probe substrate [^3^H]-l-proline^38^ at various conditions. As seen in Figure 2A, the uptake of [^3^H]-l-proline was linear up to the studied 60 min. Therefore, in the following experiments, the uptake time was selected as 60 min to gain the maximum detected amount into the cells, also with smaller concentrations. The concentration-dependent uptake experiment (at 10–600 μM concentrations) showed that [^3^H]-l-proline was effectively uptaken into the cells, having *V*_max_ of 1.13 ± 0.15 nmol/min/mg protein and *K*_m_ 1467 ± 257 μM (Figure 2B). The uptake of [^3^H]-l-proline was also Na^+^-dependent and significantly reduced in the absence of sodium (Figure 2C). The uptake of [^3^H]-l-proline showed also significant pH dependency (Figure 2C). Last, the unselective SNAT ligand, MeAIB, was able to inhibit the uptake of [^3^H]-l-proline at 50 and 100 μM concentrations (Figure 2D). However, at lower concentrations of [^3^H]-l-proline (25 μM), the inhibition by MeAIB was negligible, most likely because [^3^H]-l-proline can also use other transport mechanisms, such as proline transporter PROT (SLC6A7), in addition to SNATs that were inhibited.

### Transporter-mediated Uptake of Compounds into Cells

**Figure 3:**
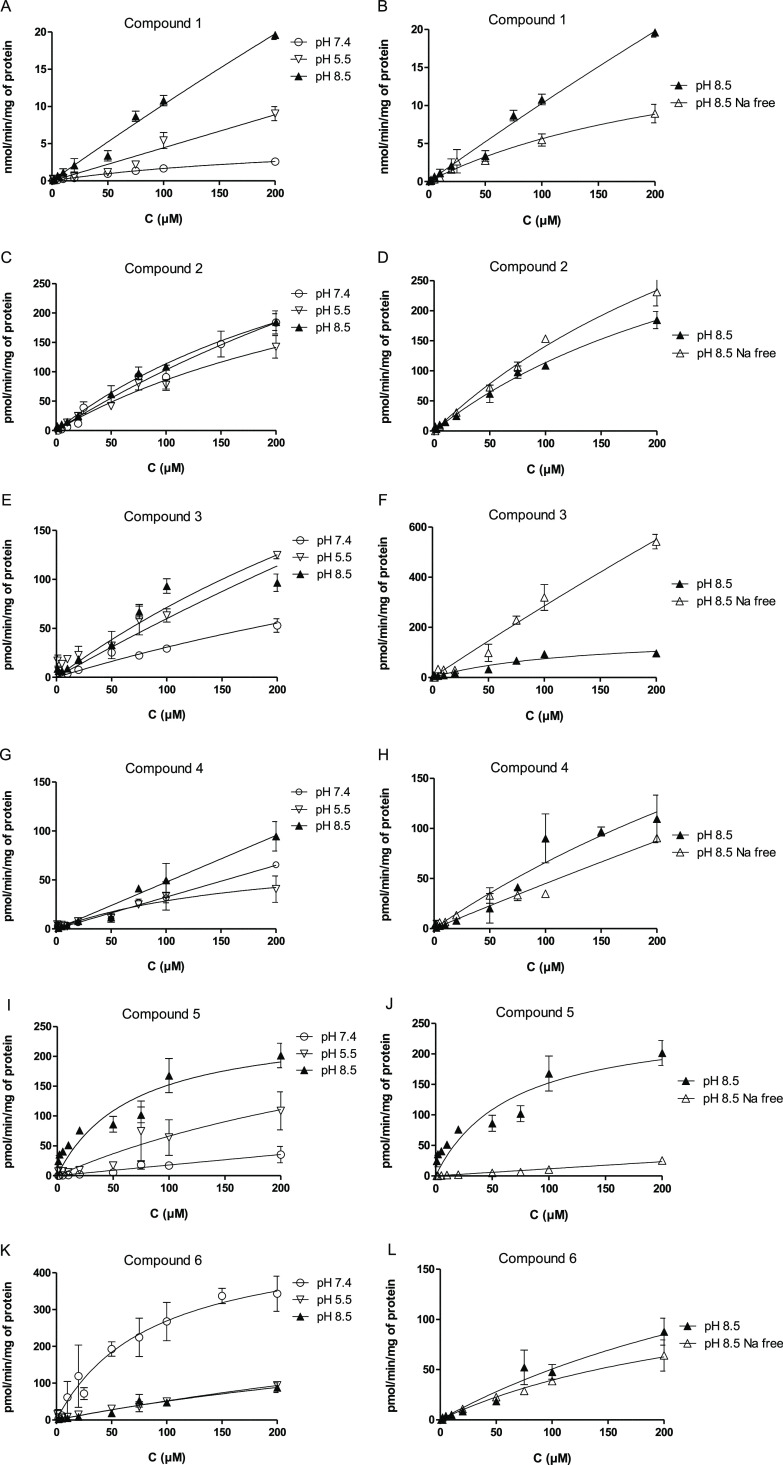

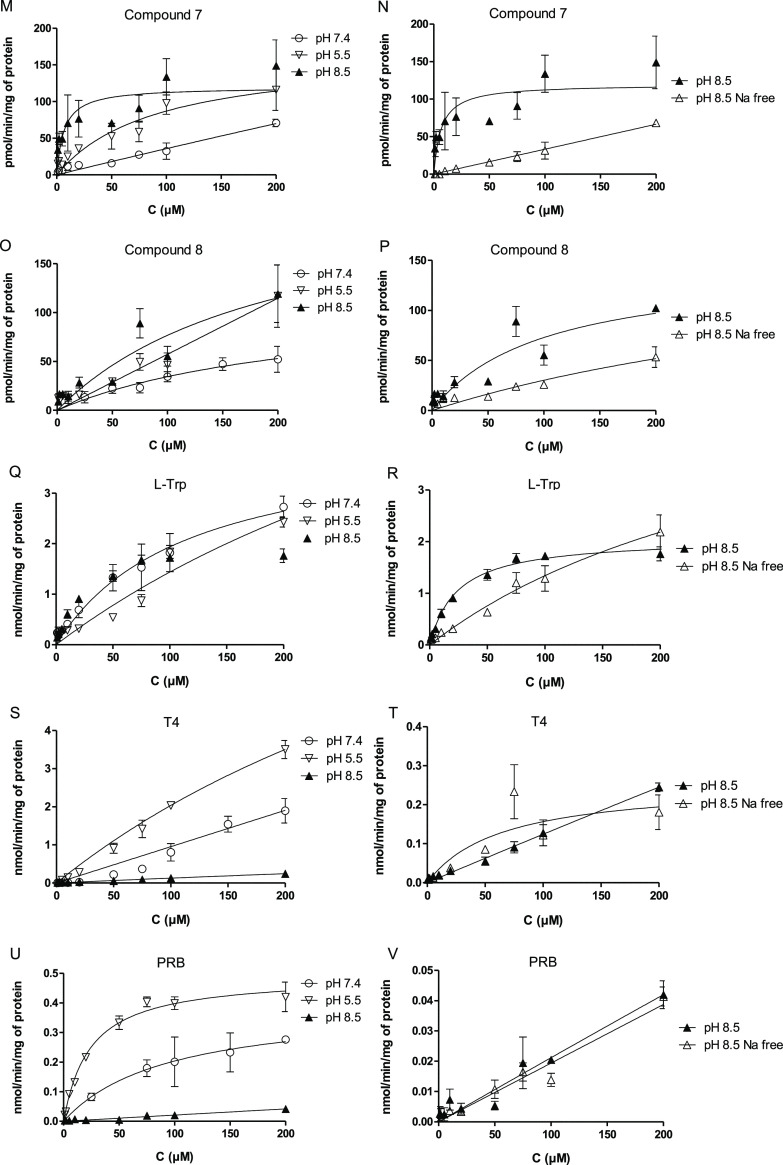
Cellular uptake of compounds **1–8**, l-Trp, T_4_, and PRB (1–200 μM) into MCF-7 cells at various pH [5.5 (open down-facing triangle), 7.4 (○ open circle), 8.5 (▲ filled up-facing triangle); left side column] and pH 8.5 in the presence (▲ filled up-facing triangle) and absence (Δ open up-facing triangle) of sodium (right side column). Note that the uptake of compound **1**, l-Trp, T_4_, and PRB is expressed in nmol/min/mg protein, while the ones of compounds **2–8** are expressed as pmol/min/mg protein. The results are expressed as mean ± SD, *n* = 3.

The cellular uptake of the studied compounds (Table 1) into human breast cancer MCF-7 cells was studied at pH 5.5, 7.4, and 8.5 and at pH 8.5 in the presence and absence of sodium to see if the transporter-mediated delivery was affected by the changes in the medium. Compound **1** has been previously shown to interact with OATPs in addition to LAT1 since the presence of LAT1 and OATP inhibitors (rifampicin and naringin) decreased its cellular uptake. The present results proved that there were interactions with acidic sensitive transport mechanisms, like OATPs (Figure 3A); the cellular uptake of compound **1** into MCF-7 cells was 3.9 times higher (*V*_max_ 29 nmol/min/mg protein) at pH 5.5 compared to the one at pH 7.4 (*V*_max_ 7.4 nmol/min/mg protein). However, the cellular uptake at pH 8.5 was even greater, 9.3 times higher (*V*_max_ 69 nmol/min/mg protein), and this mechanism was dependent on the sodium ions since the absence of Na^+^ in the medium decreased the cellular uptake at pH 8.5 (Figure 3B). Therefore, it was hypothesized that this tertiary mechanism could be mediated via one of the SNAT members. A similar pattern was observed also with compounds **4**, **5**, **7**, and **8** (Figure 3G–J,M–P). Notably, the affinities of these compounds measured as *K*_m_ values in different pHs overlapped to some extent, 1–54 μM at pH 7.4 (LAT1-mediated uptake), 47–372 μM at pH 5.5 (OATP-mediated uptake), and 40–412 μM at pH 8.5 (SNAT-mediated uptake). This implies that these LAT1-utilizing compounds are not selectively utilizing only LAT1 for their cellular accumulation.

Contrarily, the differences in the cellular uptake among non-physiological pH and the absence of sodium ions did not affect the cellular uptake of compound **2** or l-Trp (Figure 3C,D,Q,R). Curiously, the cellular uptake of compound **3** was higher at both pH 5.5 and 8.5 than at pH 7.4, and the uptake at pH 8.5 was increased in the absence of Na^+^ ions (Figure 3E,F), implying the involvement of some other alkaline-sensitive but sodium-independent transport mechanism. Furthermore, compounds **6**, T_4_, and PRB showed decreased cellular uptake at pH 8.5 compared to pH 7.4 (Figure 3K,S,U). However, since PRB and T_4_ are OATP substrates, their uptake was also increased at pH 5.5, unlike compound **6**. Nevertheless, none of these compounds reacted to the absence of Na^+^ ions (Figure 3L,T,V). Thus, based on these concentration-dependent uptake studies that may involve several transport mechanisms, no direct conclusions can be drawn as the other transport mechanisms may compensate for the missing ones in extreme conditions, such as in the absence of sodium ions.

### Interactions of Compounds with Sodium-dependent Transporters

**Figure 4:**
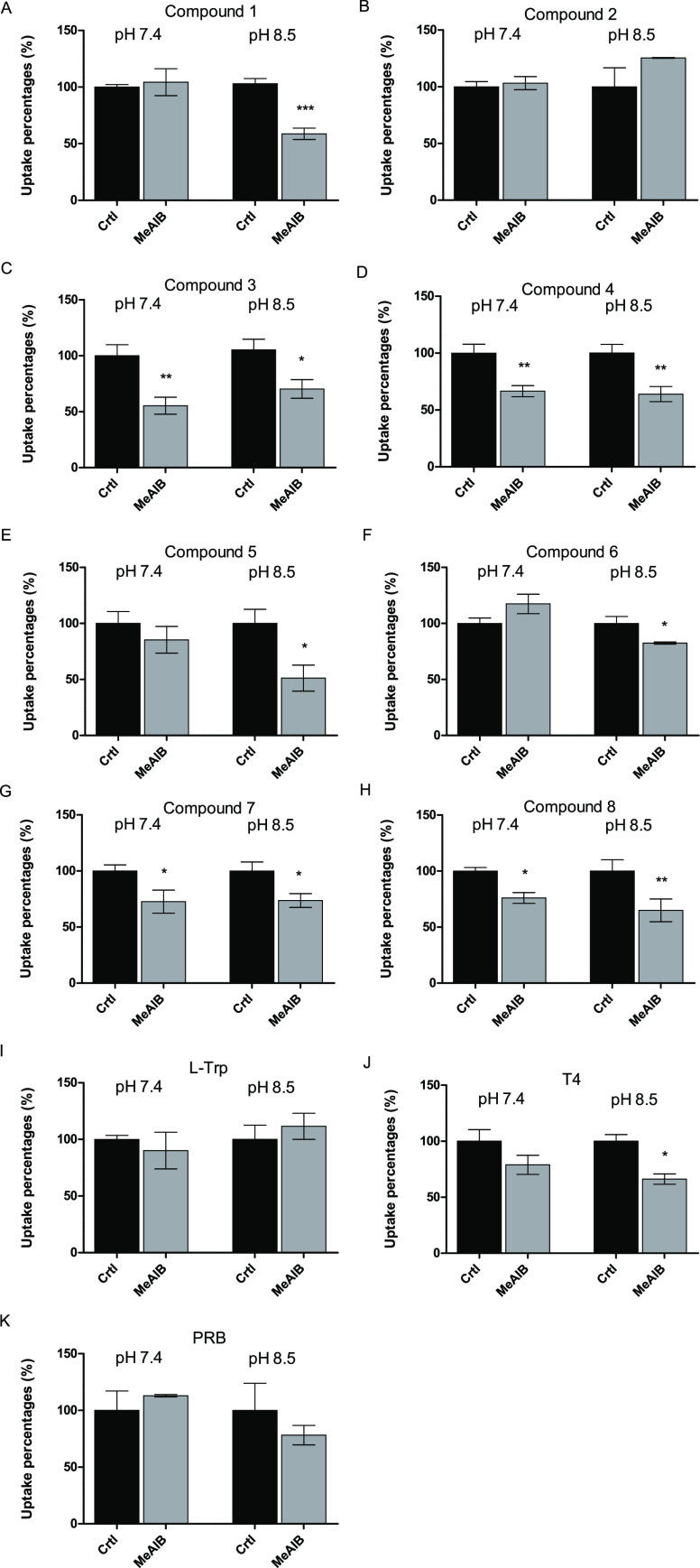
Cellular uptake of 100 μM compounds **1–8**, l-Trp, T_4_, and PRB in the absence and presence of the unselective SNAT ligand, MeAIB (100 μM). The results are expressed as mean ± SD, *n* = 3. The inhibition of cellular uptake of each compound is compared to the cellular uptake without the inhibitor (Crtl) and expressed as percentages (%). An asterisk denotes a statistically significant difference from the respective control (*P < 0.05, **P < 0.01, and ***P < 0.001, one-way ANOVA, followed by Tukey’s multiple comparison test).

To evaluate whether the studied compounds can truly utilize SNATs for their cellular internalization, the compounds were incubated together with a known system A (SNAT1–2, 4) competitive substrate (or so-called inhibitor), MeAIB.^31−33^ As seen in Figure 4A,C–H,J, compounds **1**, **3**, **4**, **5**, **6**, **7**, and **8** as well as T_4_ had interactions with SNATs as their cellular uptake was decreased in the presence of MeAIB, particularly at pH 8.5. These results were in accordance with the cellular uptake studies, in which the elevated pH increased compounds’ accumulation into the cells, and contrarily, the absence of sodium ions decreased it (Figure 3). The only exception was compound **3**, whose uptake was increased in the absence of Na^+^ ions at pH 8.5, which does not relate to the activity of SNATs. However, the cellular uptake of compound **3** at pH 8.5 was higher than that at pH 7.4, and therefore, there may be another high-capacity transport mechanism taking over when SNAT-mediated uptake is hindered due to the absence of Na^+^ ions. This would explain the contradiction between these MeAIB-inhibition and concentration-dependent uptake studies. However, this needs to be interpreted carefully, and based on these data, the possible interactions of these compounds with SNATs cannot be excluded (an inhibition trend can be seen at pH 8.5).

As expected, the presence of MeAIB in either pH 7.4 or 8.5 did not affect the uptake of PRB, l-Trp, or surprisingly compound **2** (Figure 4B,I,K). Thus, no conclusions could be drawn from the fact whether the compounds were “good LAT1-substrates” (compounds like **1** and **2**) or “poor LAT1-substrates” (compounds like **5** and **6**) and if that would have effects on the interactions with SNATs (Table 1). Moreover, no differences were observed among different promoieties attached to the parent drugs; compounds **1–4** and **7–8** have an aromatic amino acid residue as a promoiety, while compounds **5–6** have aliphatic amino acid promoieties. To understand the chemical features of LAT1-utilizing compounds that support interactions with the most abundantly expressed SNAT member, SNAT2, in the MCF-7 cells, molecular modeling and MD simulations were carried out in the next phase of the study.

### Potential pH-dependent Binding Mode of Prodrugs

**Figure 5:**
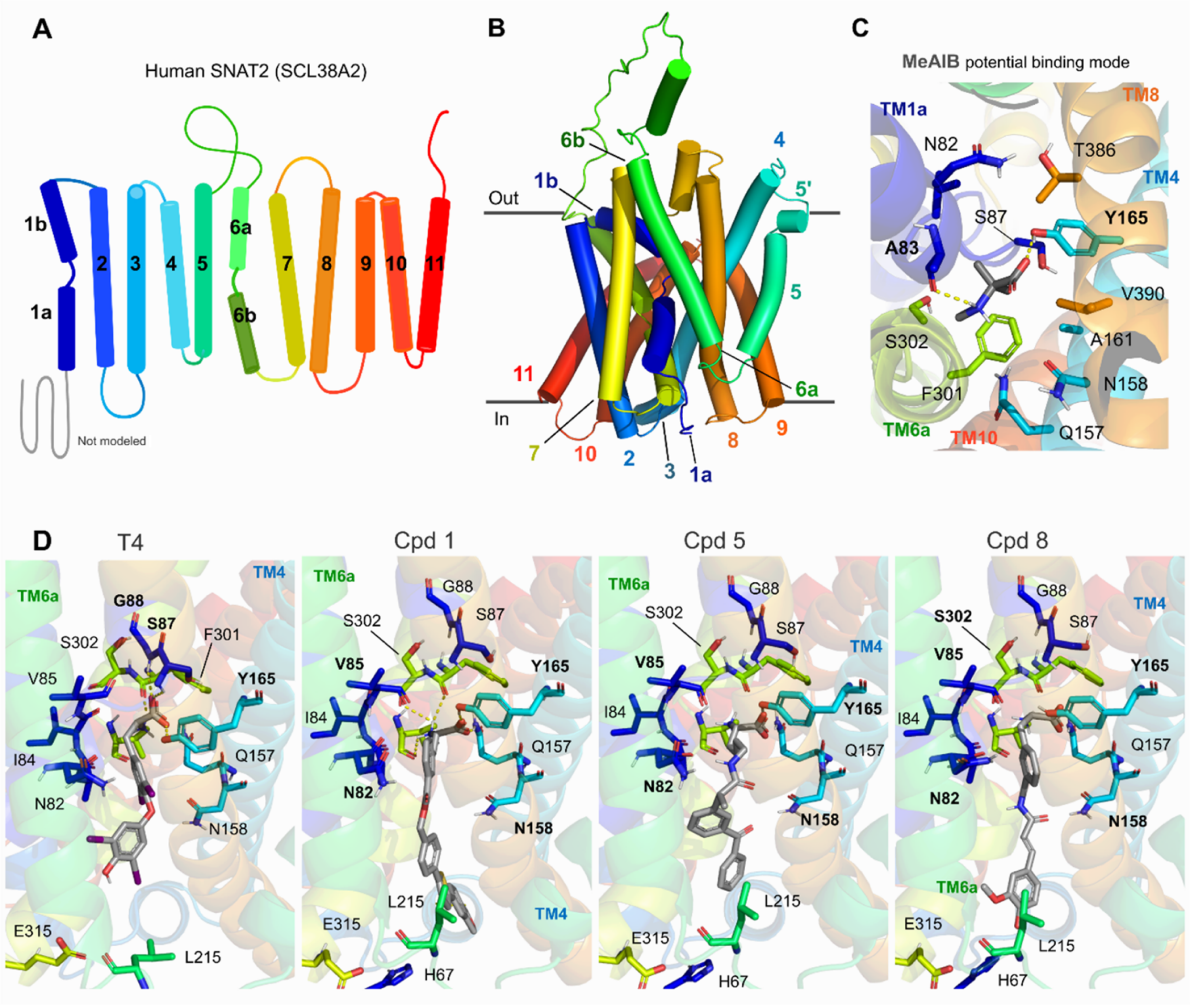
A 2D-schematic representation of the SNAT2 helix topology (A) and its respective homology model (B). Potential binding site generated from representative MD frames for MeAIB, derived from the superimposition against *Dr*SLC38A9 structure as an initial hit (C), followed by the interactions of T_4_, and compounds **1**, **5**, and **8** (D).

Members of the SLCA38 family share an extended N-terminal soluble domain and 11 transmembrane helices (TMs 1–11 with 1a and 1b as well as 6a and 6b), which were captured by the SNAT2 model (Figure 5A,B) available in the AlphaFold database. Our model resembles the so-called inward-open conformation, with TM1, TM6a, and TM7 open toward the cytosol. Due to the low confidence, the first 64 amino acids from this model were removed from further analyses and capped. This model agrees with previously proposed SNAT2 homology models based on the cryogenic electron microscopy (cryo-EM) *Danio rerio* SLC38A9 (arginine transporter^50^) and the proposed topology for rat SNAT2,^51^ with the N-terminus of SNAT2 located intracellularly and the C-terminus pointing toward the extracellular.

It is interesting to highlight that our model maintained the proposed disulfide bond between Cys245 and Cys281. However, a deeper investigation of the effects of N-glycosylation sites on the loop dynamics was out of the scope. This model was used to generate a potential binding mode for MeAIB using flexible docking, followed by MD simulations, in order to study the stability of the amino acid interactions. MeAIB fitted in a pocket composed of amino acids from the inner surface of TM1a (Ala83, Asn82, and Ser87) and TM6 (Phe301) as well as TM4 (Tyr165, Gln157, and Asn158). The carboxylate group of MeAIB mainly interacted with the side chain from Tyr165, Ser87, and Asn158 (>30% of the studied simulation time), while the positively charged α-amino group was stabilized by Phe301, Ala83, and Ile84’s main-chain carbonyl groups (Figure 5C and Table S2). Not surprisingly, the proposed binding mode for compounds T_4_, **1**, **5**, and **8** shared similar interaction features in the amino acid portion (Figure 5D). However, residues, such as Gln157, Asn158 (TM4), and Glu315 (TM6a), seemed to play a stronger role for the larger compounds (Figure 5D and Table S2). Among all our simulated compounds, **6**(*s*,*s*) carboxylate moiety displayed the highest interaction frequencies (>70%) with relevant residues Gln157 and Tyr165 and between its amino group and Ala83 (Table S2). Compound **6** also displayed the highest interaction frequency values at higher pH, which supports the role of pH in this transport conformation. It is important to also highlight that the amino and carboxylate moieties from compound **6** are responsible for the main interactions, which are unaffected by the pH and common for several transporters. This suggests that our current working model can corroborate the binding to transporters, but a fine discussion on selectivity would require more sophisticated binding energy analyses.

The cryo-EM structure *Dr*SLC38A9 (PDB ID: 6C08) is displayed in a cytosol-open state, where the arginine remains stabilized by interactions with TM1a, TM3, and TM8.^52^ In agreement with our model, the arginine’s carboxylate interacts with Tyr204 (TM3) and Gln438 (TM8), while the α-amino group has hydrogen bond interactions with the Thr121 and Ser122 (TM1a) and is further stabilized by Tyr204 (TM3).

**Figure 6:**
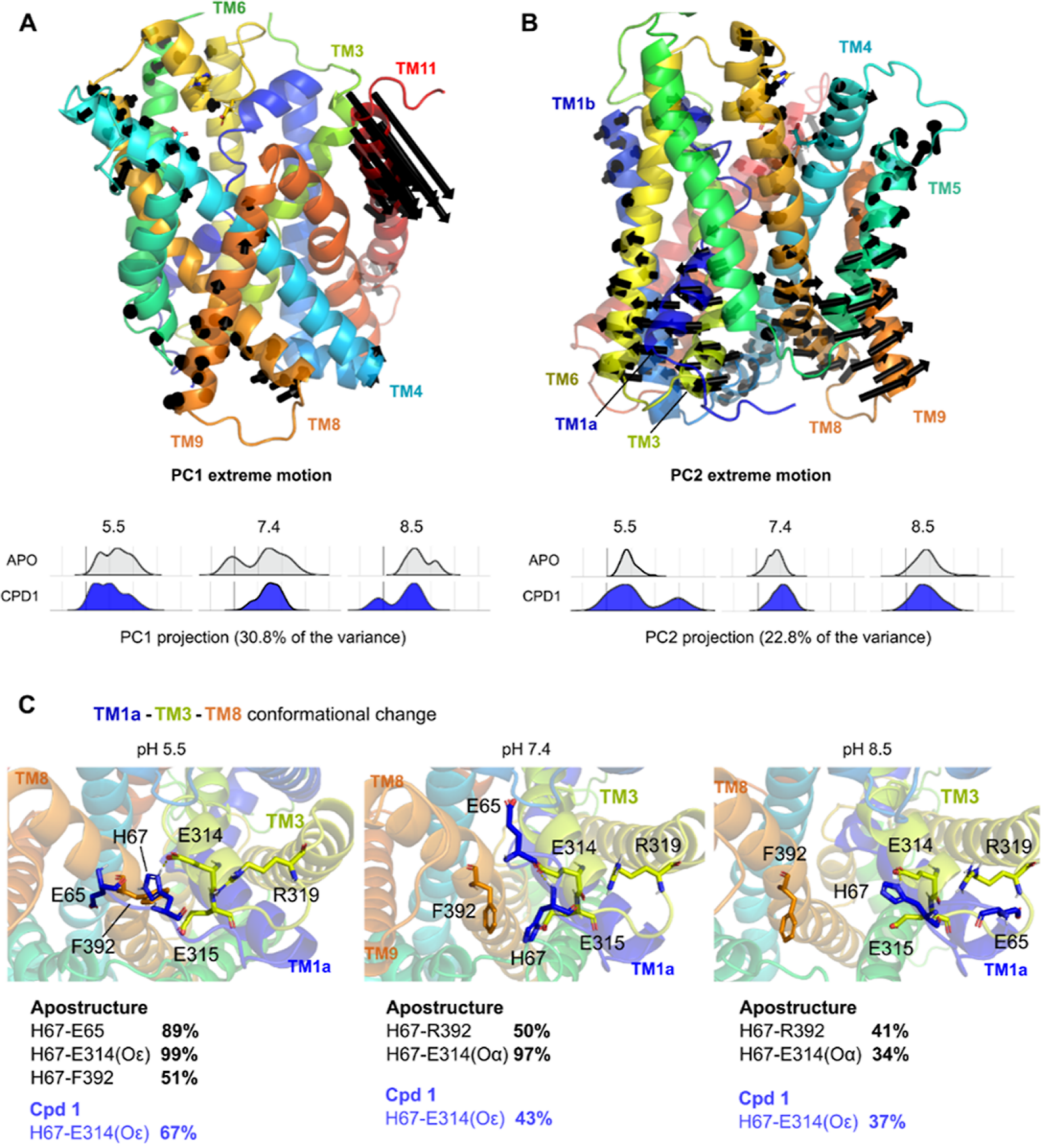
Extreme motions from PC1 (A) and PC2 (B) displayed over the SNAT2 model represented by dark arrows. The distributions (A,B below) over the two significant PCs [PC1 (A) and PC2 (B)] are separated for each simulated system apostructure (gray) and compound **1** (blue). Representative conformational changes of SNAT2’s inward cavity due to different pHs, followed by the interaction frequency between relevant pairs of amino acids (C). SNAT helices are colored according to Figure 5.

Interestingly, more frequent interactions and often different patterns were observed in simulations at pH 5.5 and 8.5, in comparison to pH 7.4, such as the interaction of compound **8** with Ile215, even though none of the ligand-binding amino acids changed their ionization state upon the pH change. This prompted us to systematically analyze our MD trajectories, combining all the different pHs and compounds, by using PCAs. Since the principal components with the highest eigenvalue are those that explain the majority of the data variability, in this case, our PC1 (30.8% of the variability) and PC2 (22.8%), we chose to analyze the associated protein regions whose motions contribute to these differences (Figure 6A,B). PC1 projection did not show a distinction between different pH values or among the tested compounds. This suggests that protein regions depicted by the PC1 extreme motion vectors, showing a clear change in the TM11 (in red, Figure 6A) in the outward-facing part of the transporter, are not relevant for pH modulation. Curiously, the PC2 projection contribution (Figure 6B) suggests that the pH conformational changes, initially observed in the apostructure, are more pronounced in the inhibited state (as illustrated by the PC2 of compound **1** at pH 5.5). Its associated PC2 extreme motion displayed the concerted opening of TM1a/TM3 (blue and lime colors in Figure 6B) away from TM8/TM9 (depicted with orange in Figure 6B), which were further investigated. Double ionization of His67 (TM3) at pH 5.5 and consequently its positive charge allowed the formation of salt bridges with Glu65’s side chain (TM1a, by 99% in apostructure simulations) (Figure 6C). This interaction was either absent at higher pH-values, such as in apostructure simulations (as depicted with black in Figure 6C and respective Table S3), or less frequent, such as in compound-bound simulations (for instance, trajectories of compound **1**; blue in Figure 6C), since the side chain of the prodrugs occupied the space between these helices. It is important to highlight that during our simulations, a classical force-field model was adopted, which disregards potential changes in the ionization during the calculation. This brings relevant implications for the histidine side chains, which could further display ionization changes due to the polarization effect from interactions with other amino acids.

**Figure 7:**
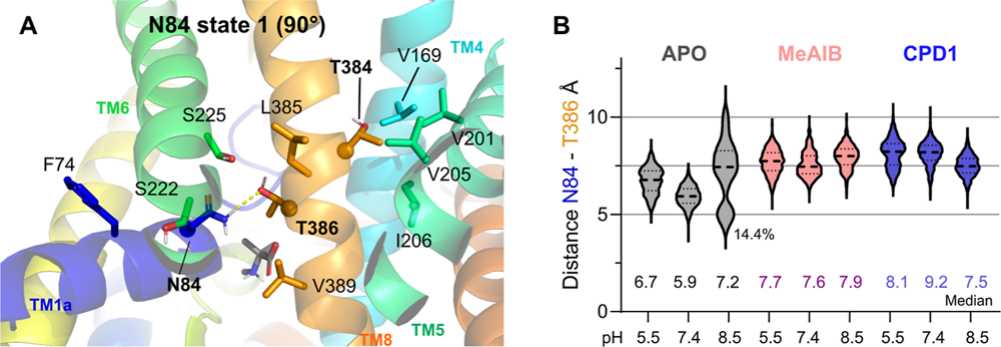
(A) Inset on the SNAT2 potential salt binding motif (residues highlighted in bold and displayed as spheres). (B) Distances between the center of mass of Asn84 and Thr386 were calculated along the trajectory time. Median distance values for the distributions are provided in the bottom figure, and for the apostructure at 8.5, the smaller distribution range frequency is displayed.

The work from Zhang 2008 and 2009^53,54^ shows that Thr384Ala (TM8) and Asn82Ala mutation inhibits the anion leak current and lowers the Na^+^ affinity of SNAT2, while Asn82Ser (TM1) displays a less dramatic effect. Overall, it suggests that these two residues would compose a Na^+^ binding site; however, our models show no direct interaction between Asn84 and Thr384 (which are at least 12 Å apart). Thus, we chose to investigate the connection intermediated by Thr386 interaction (composing a TLT motif, Figure 7A). The Asn82 side chain displayed two main conformations along the simulation trajectories that correlated with the torsion of helix TM1. In the first conformation, Asn82 side-chain (Figure 7A,B) amide group interacted with the hydroxyl from Thr386 (TM8), and this conformation locked Thr384 in the position where its methyl group occupied a hydrophobic pocket composed of Val169 (TM4), Val201, Val205, and Val206 (TM5). This conformation was more accessed by the apostructure simulation (Figure 7B). Meanwhile, the second Asn82 conformation occurs with torsion of TM1, allowing its side chain to display water-mediated interactions with Thr386. This conformation was more often accessed by the inhibited state (Figure 7B and Table S3).

The Asn84–Thr386 polar interactions were often water-mediated and not very frequent (∼30% of the simulation time, Table S3). Furthermore, it was observed to be disrupted by our compounds (Figure 7B). This conserved water site is proposed by triangulating the water-mediated hydrogen bonds between the side chains of Asn84, Ser222, Ser225, and Thr386, and it is relevant as a potential sodium binding site. However, interestingly, none of these sites coordinate sodium ions along the studied trajectories (data not shown), which could be explained by the lack of coordinated sodium ions in the initial models, too short simulations to allow the entrance of new sodium molecules, or by the limitation of the employed force-field. It is noteworthy to mention that no ions were placed in points of potential coordination, although the simulations were conducted in the presence of sodium.

## Discussion

It is very common that compounds can have not only one but several transport mechanisms. On the other hand, many proteins, like transporters, also have overlapping substrate specificities. However, in drug development, we tend to simplify the complex phenomena of drug delivery and often look only at the most obvious transport mechanisms. LAT1-utilizing prodrugs are classical examples. We have already shown in the past that quite many amino acid derivatives (LAT1 prodrugs) have interactions with OATPs.^21^ In the present study, we have found that these compounds can also interact with SNAT-family members. Since functional SNAT2 was highly expressed in our test system, in MCF-7 human breast cancer cells (Figures 1 and 2), we focused only on the interactions of the studied compounds with SNAT2. However, it is likely that these compounds can have interactions with other SNATs too since according to the protein atlas (www.proteinatlas.org, accessed 9.5.2022), SNAT6–7 and 9–10 are also expressed in MCF-7 cells.

Notably, the expression of SNAT2 protein on the plasma membrane of MCF-7 cells was 3 times higher compared to LAT1. Therefore, the possibility to utilize this transporter if the compound has favorable structural features for SNAT2 interactions is increased. As was shown in the present study with many LAT1 prodrugs, such as compounds **1**, **4**, **5**, **7**, and **8**, the cellular uptake was indeed much higher at pH 8.5, optimal for SNATs, compared to cellular uptake at pH 7.4 (Figure 3). The cellular uptake of these prodrugs at pH 8.5 was also sodium-sensitive. It has been proposed that the extracellular C-terminal histidine residues of SNATs are pH-sensing and thus able to regulate the binding of Na^+^ allosterically and subsequently also the binding of the amino acid substrates to SNATs.^55^ Moreover, it has been estimated that at the physiological conditions (pH ca. 7.4), the capacity of SNATs is approximately in the midrange compared to their maximum capacity at pH 8.0.^55^ Therefore, these data together strongly imply that SNATs may have a significant role in the total cellular uptake of the studied compounds. However, it is difficult to estimate how strongly these secondary and tertiary mechanisms participate in physiological conditions since the affinities (*K*_m_ values) overlapped in the different pH values. Furthermore, the present in vitro studies were carried out with relatively high concentrations, and thus, the affinity of compounds to each transporter at their therapeutic window remains to be elucidated.

Curiously, SNATs mRNA and protein synthesis are strongly up-regulated by amino acid deprivation and hypertonicity, and it has been suggested that the SNAT2 stability is dependent on the substrate-induced cycling, in which the transporter exposes its cytosolic N-terminal lysine residues in a specific conformation for the ubiquitin-proteasome system, resulting in destabilization degradation of intracellular SNAT2.^56,57^ Therefore, the exact role of SNAT2 on the pharmacokinetics of its substrates within the selected period of time is overall very challenging to estimate. Moreover, it is well known that LAT1 is expressed on the lysosomal membranes and we have also shown that LAT1-utilizing compounds can accumulate into the lysosomal cell fraction more effectively than their parent drugs,^35,58^ while not that much is known about the trafficking of SNAT2 between the intracellular and plasma membranes. Thus, the role of SNAT2 in the intracellular compartmentalization of amino acids and their derivatives is even more difficult to estimate than its role in total cellular uptake at physiological conditions.

Unfortunately, no trend between the affinities for LAT1 and SNAT2 was found. Thus, the lesser interactions with LAT1 (higher IC_50_ and CL_int_ values in Table 1) did not correlate with greater SNAT2 interactions. In addition, the overlapping transport mechanisms also complicated the interpretation of the concentration-dependent cellular uptake studies and the Michaelis–Menten kinetics (linear and not clearly saturating uptake in Figure 3). Moreover, the uptake inhibition studies of the selected compounds with the unselective SNAT ligand, MeAIB, which should indicate the SNAT2-mediated transport, were not clear for all the studied compounds, particularly at pH 7.4 (Figure 4). In many cases, MeAIB inhibited the uptake of studied compounds only at pH 8.5. Thus, the other interactions (possibly with OATPs) at pH 7.4 complicated the situation as they can compensate for the inhibited SNAT2-mediated proportion of the total uptake, and therefore, no inhibition with MeAIB at pH 7.4 was seen. Furthermore, due to the possible regulation of substrate binding in the absence of sodium Na^+^, the results of concentration-dependent uptake studies in the absence of Na^+^ at pH 8.5 were not consistent with inhibition studies with MeAIB at pH 8.5 (like in the cases of prodrugs **3**, **5**, **6**, **7**, and T_4_). Therefore, this study highlights that inhibition experiments should be carried out not only in physiological conditions but also in extreme conditions, such as a slightly acidic environment (pH 5.5. increases OATP interactions) and slightly elevated pH (supporting SNAT-interactions), as well as in the presence and absence of sodium ions to understand the determinants affecting the possible interactions with secondary and tertiary transport mechanisms, particularly when working with native cells (Table 2). Alternatively, to reveal the real utilization of a specific target transporter, genetically modified cells, such as transporter-transfected cells or silencing the target transporter by siRNA, could have also been used. However, in the present study, the overall aim was to understand which chemical features can predispose these LAT1-utilizing compounds for the interactions with SNAT2, and therefore, molecular modeling approach was used to give more insights on this phenomenon.

**Table 2:** Summary of the Interactions of the Studied Compounds **1–8**, l-Trp, T_4_, Probenecid (PRB), and α-(Methylamino)-isobutyric Acid (MeAIB) with Selected Transporters (LAT1, OATP1C1, and SNAT2) Based on the Cellular Uptake Studies at Different pHs with MCF-7 Cells

compound	LAT1 (pH 7.4)	OATP1C1 (pH 5.5)	SNAT2 (pH 8.5)
**1**	+++	+++	+++
**2**	+++		
**3**	++	+	+
**4**	++	+	+
**5**	+	++	+++
**6**	+		+
**7**	++	++	++
**8**	+	++	++
l-Trp	+++	+	
T_4_	++	++	+
PRB		+++	
MeAIB			+++

Thus, to generate the potential binding mode for studied compounds, a SNAT2 3D-structure model derived from the AlphaFold database was used. The used model resembles the inward-open conformation with a large central cavity composed of the TM1, TM3, and TM8. Analyses of the simulation trajectories underscored the relevance of pH in the transition between the inward- and outward-open states. The current SNAT2 model was able to propose a consistent binding mode for the studied compounds, mainly relying on the interactions between the amino acid portion and conserved residues in the aforementioned helices. However, it falls short of fully describing the binding strength since a poorer SNAT2 compound, T_4_, showed similar binding features as the good SNAT2 ligands, like compounds **1** and **8**. One of the strongest SNAT2 binders, compound **6**(*s*,*s*), displayed the highest interaction frequencies (>70%) with relevant residues Ala83, Gln157, and Tyr165, which we suggest composes the major features for SNAT2 binding. Moreover, compound **6** also displayed the highest interaction frequency values in higher pHs, which supports the pH’s role in this transport conformation.

Given the relevance of sodium in the cotransport mechanism of SNATs, we shifted our attention to discussing the conformation of the sodium binding sites in SNAT2 trajectories. However, despite the interesting conformational change observed for Asn84 toward Phe74, no sodium atoms coordinated to these residues were observed. Instead, we found a conserved hydration site encompassing Asn84, Ser222, Ser225, and Thr386 side chains. It is important to mention that the identification of sodium binding sites, using structural biology, remains a challenge due to the high resolution that is required. In order to differentiate between the electron densities of sodium and water in crystals, it is necessary to resolve structures greater than 1.2 Å.^59^

Additionally, previous simulations with betaine transporters (BetP), starting from the inward-occluded conformation, suggested that possible sodium binding sides (pNa1 or pNa2) could not stably coordinate the sodium ions.^60^ However, in the same work, experimental evidence supported BetP binding to two ions, where the binding involved two hydroxyl side chains originating from the same face of a transmembrane helix. MD simulations of inward-facing conformations of vSGLT showed that ions, placed prior to simulation, are released from the proposed binding site,^61,62^ and inward-facing vSGLT structures showed no electron densities for sodium ions in the conserved Na1 sites. Overall, this suggests that despite experimental evidence, MD simulations of inward conformations are unable to properly capture sodium coordination.

Thus, even though we were able to experimentally identify compounds that can have interactions with SNATs, only the more detailed MD simulation analyses revealed the relevant interactions of the studied compounds with SNAT2. However, due to the small number and similarity of the compounds, no exact structure–activity relationships can be drawn from these data. Hence, the most important conclusion of the present study is that the compounds that are originally designed toward some other transporter, such as LAT1 in this case to improve their brain drug delivery, may have interactions with secondary and tertiary transport mechanisms, like OATPs and SNATs (Table 2). Therefore, these interactions may have a huge impact on their pharmacokinetic profile and distribution to specific tissues, like the brain. Thus, it is very important to also look at other interactions that the studied compounds may have, including the interactions with efflux transporters, which may limit the prodrug exposure in the desired tissues and cells. Most importantly, it would be highly fundamental to work not only with the genetically modified cells but also with the real target cells of the compounds and understand their transporter expression profiles and transporting mechanisms, that is, the pharmacoproteomics that ultimately determine the pharmacological effects of the studied compounds.

These secondary and tertiary mechanisms can have positive and delivery-increasing effects on the compounds to the specific tissues or cells, but they can also have negative and off-target increasing effects. SNAT2 is expressed in the brain but also in other tissues and it is overexpressed in several cancer types,^25,29,63,64^ and therefore, predicting its role in the pharmacokinetics of LAT1-utilizing compounds is challenging. When comparing the findings of the present study to the pharmacokinetic studies and accumulation of these prodrugs into the brain that have been reported previously (Table 1), no clear correlation on how these additional mechanisms could affect was found [*K*_p_ values (AUC_brain_/AUC_plasma_) were on the same level; 0.017–0.082, the only exception was compound **6**; 0.317]. Nevertheless, the effects of other transport mechanisms may become even greater if the primary transporter becomes unfunctional or down-regulated due to specific conditions, such as in diseases, or due to the polymorphism that some transporter may have. Moreover, it is also very important to pay attention to how environmental factors, including physical, chemical, and biological factors, can affect the expression and function of primary, secondary, and tertiary transport mechanisms in future studies. After all, the non-primary transport mechanisms may be the main determinants of pharmacokinetics and thus have a huge effect on the drug disposition and clinical outcome of drugs.

## Conclusions

In a summary, the present study shows that amino acid-drug conjugates intended to utilize primarily LAT1 can have interactions with other transporters, like SNAT2, in addition to previously reported OATPs. These secondary and tertiary transport mechanisms can have a major impact on these compounds’ cellular uptake. This may affect the pharmacokinetic profiles and particularly the targeting effect of these compounds, for example, into the brain or tumor sites, which are often the sites of action of LAT1-utilizing compounds. Therefore, it is highly important to screen additional interactions of novel compounds toward several distinct transporter mechanisms to attain more reliable translation from in vitro to in vivo and from rodent to human situations.

## Supporting Information Available

SRM/MRM transitions for absolute quantitative proteomics, protein–ligand polar interaction frequency during the analyzed trajectory for each compound, and protein–protein interaction frequency during the analyzed trajectory for each compound (PDF)
Molecular formula strings (XLSX)

## Notes

PDB ID codes: PDB code 6C08 was used for the l-arginine-bound *Dr*SLC38A9 structure homology model.

## Notes

Homology models: AlphaFold identifier AF-Q96QD8-F1 was used for SNAT2 3D-structure model (SLC38A2 UniProt ID: Q96QD8).

## Abbreviations

ABC: ATP-binding cassettes
BBB: blood–brain barrier
EMA: European Medical Agency
FDA: U.S. Food and Drug Administration
GLUT1: glucose transporter 1
LAT1: l-type amino acid transporter 1
MCF-7: human estrogen receptor-positive breast adenocarcinoma cell line (Michigan Cancer Foundation-7)
MD: molecular dynamics
MeAIB: α-(methylamino)isobutyric acid
OATP: organic anion transporting polypeptide
OBLS: *O*-benzyl-l-serine
PRB: probenecid
SLC: solute carriers
SNAT: sodium-coupled neutral amino acid transporter
T_4_: thyroxin
TM: transmembrane
