# Supplementary material for: Sodium-Dependent Neutral Amino Acid Transporter 2 Can Serve as a Tertiary Carrier for l-Type Amino Acid Transporter 1-Utilizing Prodrugs
Source: Mol Pharm. 2023 Jan 23;20(2):1331–46. doi: 10.1021/acs.molpharmaceut.2c00948 (PMC9906736; doi:10.1021/acs.molpharmaceut.2c00948)
Supplement: Supplementary file 1 — mp2c00948_si_001.pdf [file mp2c00948_si_001.pdf]

Supplementary Information

Sodium-dependent Neutral Amino Acid Transporter 2 (SNAT2) Can Serve As A Tertiary Carrier for L-Type Amino Acid Transporter 1 (LAT1)-Utilizing Prodrugs

Johanna Huttunen [a], Thales Kronenberger [a,b], Ahmed B. Montaser [a], Adéla Králová [a], Tetsuya Terasaki [a], Antti Poso [a], Kristiina M. Huttunen [a,]*

[a] School of Pharmacy, Faculty of Health Sciences, University of Eastern Finland, P.O. Box 1627, FI-70211 Kuopio, Finland;

[b] Department of Internal Medicine VIII, University Hospital Tuebingen, Otfried-Müller-Strasse 14, Tuebingen DE 72076, Germany. b Department of Pharmaceutical and Medicinal Chemistry, Institute of Pharmaceutical Sciences, Eberhard-Karls-Universität, Tuebingen, Auf der Morgenstelle 8, 72076 Tuebingen, Germany.c Cluster of Excellence iFIT (EXC 2180) "Image-Guided and Functionally Instructed Tumor Therapies", University of Tuebingen, 72076 Tuebingen, Germany. d Tuebingen Center for Academic Drug Discovery & Development (TüCAD2), 72076 Tuebingen, Germany

* Corresponding author, E-mail: kristiina.huttunen@uef.fi

Contents

Table S1. SRM/MRM transitions for absolute quantitative proteomics (Uchida et al., 2015, Uchida et al., 2020)....................2

Table S2. Protein-ligand polar interaction frequency during the analyzed trajectory for each compound....................3

Table S3. Protein-protein interaction frequency during the analyzed trajectory for each compound. 4

Supplementary Information

Table S1. SRM/MRM transitions for absolute quantitative proteomics (Uchida et al., 2015, Uchida et al., 2020).

Protein	Gene	Peptide	Type	Retention time (min)	Precursor ion Q1	Product ions Q3-1	Q3-2	Q3-3
Na^+K^+ATPase	*ATP1A1-3*	AAVPDAVGK	St	10.3	414.23	685.39	586.32	489.27
		AAVPDAVG**K***	SIS	10.3	418.24	693.40	594.33	497.28
LAT1	*SLC7A5*	VQDAFAAAK	St	12.7	460.75	693.36	578.33	821.42
		VQDAFA**A***AK	SIS	12.7	462.75	697.36	582.34	825.42
GLUT1	*SLC2A1*	TFDEIASGFR	St	29.2	571.78	894.43	537.28	650.36
		TFDEI**A***SGFR	SIS	29.2	573.78	898.44	541.29	654.37
SNAT1	*SLC38A1*	NELPSAIK	St	16.0	436.25	628.40	515.32	418.27
		NELPSA**I***K	SIS	16.0	439.76	635.42	522.34	425.28
SNAT2	*SLC38A2*	AFGLVGK	St	20.8	346.21	473.31	620.38	416.29
		AFG**L***VGK	SIS	20.8	349.72	480.33	627.40	423.30
SNAT4	*SLC38A4*	YELPEVIR	St	30.7	509.78	613.37	726.45	855.49
		YELPEV**I***R	SIS	30.7	513.29	620.38	733.47	862.51
SNAT5	*SLC38A5*	IVPSEVEPFLSWPK	St	47.6	814.44	874.48	1003.52	
		IVPSEVEPFLSWP**K***	SIS	47.6	818.45	882.50	1011.54	

Table S2. Protein-ligand polar interaction frequency during the analyzed trajectory for each compound. Frequency is displayed as (%) of the hydrogen bond or water-mediated interactions and separated according to the compound moiety performing it.

	**pH**	**5.5**	**7.4**	**8.5**	**5.5**	**7.4**	**8.5**	**5.5**	**7.4**	**8.5**	**5.5**	**7.4**	**8.5**	**5.5**	**7.4**	**8.5**	**5.5**	**7.4**	**8.5**	**5.5**	**7.4**	**8.5**
	**Cpd**	**MeAIB**			**T4**			**CPD1**			**CPD5**			**CPD8**			**CPD6sr**			**CPD6ss**		
Carboxylate	N82	16	3																			
	G88	37						25			90	80	41									
	S87	58	24		44	41		26			91	77			42	37						
	Q157		27		82	58	92	32	43				34	74	38	52	62	67	71	92	94	94
	N158		32		14	10		23	31	73			32	33				46	61	34	69	45
	Y165	31			52	36	75	27			39	66	51		50			65	57	98	75	66
	Y339			31																		
Amino	A83		67		43	57		80	31	46	81	64	59	54	55	48	60	80	66	94	73	60
	I84	55	25	32	20	45	36	37			55	65	31		32					32		
	V85	13						21			4			37		31	48		31			
	F301	39	58	32	45	36	79	28			35	54	54		47			34		66		
	S302				54	43	48		43		51	35	55			50	49	77	67	51	78	70
	V304		32		36	32	40	42	33	73			38	64		34	66	73	85		92	90
	H67													81								
Side chain	N79																36					37
	N82									54							45	34	35	62		47
	N158										31	90	60		55							
	L215													56		53						
	E315	12			32					62				40		30						

Table S3. Protein-protein interaction frequency during the analyzed trajectory for each compound. Frequency is displayed as (%) of the hydrogen bond or water-mediated interactions and separated according to the compound moiety performing it. Interactions performed with the side-chain of the main amino acid (His67 and Asn82) are labelled with M, while interactions with their sidechains are labelled with S. Water-mediated interactions are labelled with W.

	His67 (HIP - pH 5.5, HID - pH 7.4 and 8.5)																							
**pH**	**5.5**	**7.4**	**8.5**	**5.5**	**7.4**	**8.5**	**5.5**	**7.4**	**8.5**	**5.5**	**7.4**	**8.5**	**5.5**	**7.4**	**8.5**	**5.5**	**7.4**	**8.5**	**5.5**	**7.4**	**8.5**	**5.5**	**7.4**	**8.5**
**CPD**	**MeAIB**			**T4**			**CPD1**			**CPD5**			**CPD8**			**APO**			**CPD6sr**			**CPD6ss**		
E65				10	25		11			12						89								
E314 wS	76	52	61	39	24	26	39	43	20	75	15	21	42	26		99		31						35
E314 wM				21	40	38	19	16	28	26	67	27	19	28	34	61	97	18	35		79	54		49
E315 s	15	20		39		20	67	12	37	13	14	10	66	28	65	16	10	20	39	39		42		
R319 wM	11		12	18				28	14			10	17	49	79	25	34	41						35
F392																51								

Asn82

**pH**	**5.5**	**7.4**	**8.5**	**5.5**	**7.4**	**8.5**	**5.5**	**7.4**	**8.5**	**5.5**	**7.4**	**8.5**	**5.5**	**7.4**	**8.5**	**5.5**	**7.4**	**8.5**	**5.5**	**7.4**	**8.5**	**5.5**	**7.4**	**8.5**
**CPD**	**MeAIB**			**T4**			**CPD1**			**CPD5**			**CPD8**			**APO**			**CPD6sr**			**CPD6ss**		
F78 wM	93	99	99	91	97	94	97	98	99	95	99	98	96	97	98	93	88	98	98	98	97	97	98	97
F78 wS			29					10		24		19				60	35	12						
S222 wS	20	51		44	41	50	38	46		29	39	49	34	14	21	11	51	33		35	39	43		
S225 wS	22	89	33	19	24	25	32	24	32	41	41	32	37	38	27	27	83	19						
L385 wS		49															49	12						
T386 wS	36	52		22	28	28	20	30	39	29	57	33	41	34	37	36	24	17				38		47
